# The occurrence of giant mesenteric cyst and adrenal ganglioneuroma in a schizophrenic male patient presenting as pseudocyesis: A case report

**DOI:** 10.1002/ccr3.3496

**Published:** 2020-11-11

**Authors:** Alireza Negahi, Fatemeh Jahanshahi, Behnaz Boozari, Alireza Sadeghipour

**Affiliations:** ^1^ General Surgery Department Rasoul Akram Hospital, Iran University of Medical Sciences Tehran Iran; ^2^ Student Research Committee Faculty of Medicine Iran University of Medical Sciences Tehran Iran; ^3^ Pathology Department, Firoozgar Hospital Iran University of Medical Sciences Tehran Iran; ^4^ Pathology Department Rasoul Akram Hospital, Iran University of Medical Sciences Tehran Iran

**Keywords:** adrenal gland, case report, ganglioneuroma, laparotomy

## Abstract

In psychological patients like our case, somatically expressed symptoms which can imply another psychological syndrome should be dealt with cautiously.

## Graphical abstract

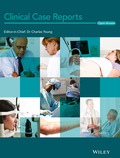

## 1 INTRODUCTION

Mesenteric cysts (MCs) are rare benign lesions found in the abdomen with an incidence rate of 1 per 100 000 adults and 1 per 20 000 children. Although it can be found at all ages, it is more common among adults at the age of between 40 and 70 years and children under 10 years without any gender preference. While their typical sizes are up to 10 cm, they can emerge in larger sizes. However, very large mesenteric cysts are rare presentations of such benign tumors.^1^, ^2^ This lesion was first diagnosed and introduced by Benineni, an Italian anatomist, in an eight‐year‐old boy, in 1507. Afterward, in 1850, Paul Jules Tillaux successfully resected a mass of this type of cyst for the first time. Subsequently, marsupialization of the cyst was conducted by Pean in 1883.^3^ The mesenteric cysts exhibit no signs in 40% of cases. They are usually diagnosed accidentally, while tumor expansion, mesenteric stretching, or pressure on surrounding organs may cause symptoms such as abdominal pain, intestinal obstruction, nausea, and vomiting.^1^

Ganglioneuromas (GNs) are benign tumors in sympathic system, originating from neural crest cells. They are composed of ganglionic cells, Shawn cells, and neurites. Generally, this tumor is found in posterior mediastinum and retroperitoneum. In addition, there are a few rare cases of its formation in the adrenal medulla. GN tumors can be found at all ages. However, the rare cases of GN tumor of the adrenal gland are more common in children and young adults. GNs are often hormonally silent and present no symptom; hence, the clinical diagnosis is difficult and virtually in all cases accidental.^4^

Both GNs in adrenal gland and giant MCs are rare phenomena. However, their occurrence in a schizophrenic patient presenting as a pseudocyesis syndrome makes the case more noteworthy. To the best of our knowledge, there has been no report on such case in the literature and this article is the first case report on this subject.

## 2 CASE REPORT

The patient was a 55‐year‐old man, who previously diagnosed with schizophrenia and had been hospitalized several times because of the disease and claimed that he was pregnant. Unfortunately, due to his mental disorder, this abnormal statement was neglected. Therefore, the schizophrenia treatment continued until abdominal pain, coffee‐ground vomit, and abdominal distension occurred. At this stage, the patient was referred to the department of general surgery. A written consent was obtained from the patient for publication of this case report and any accompanying images.

Apparently, the patient was cachectic with bilateral temporalis muscle atrophy which was not consistent with his age. Additionally, it was observed that the abdomen was evenly huge and distended. In palpation, a giant firm mass was detected in all over the abdomen. However, no mass with a definite boundary was observed in the examination. In the epigastric region, there was a mild tenderness without rebound tenderness. During the examinations, bowel sound in each quadrant of the abdomen was auscultated, and it was slightly heard in the left upper quadrant. Due to the patient's psychiatric problems, the only source of patient history was his relatives. According to their statements, the patient had experienced several times of coffee‐ground vomiting. Additionally, the patient had small bowel obstruction symptoms with low defecation frequency since a long time ago. This condition had continued until 11 days ago when there was no defecation and he had only gas passing. Since the mass was observed in the abdomen, abdominopelvic ultrasound was requested, and the result showed a cystic hypoechoic mass that had spread all over the abdomen.

**FIGURE 1:**
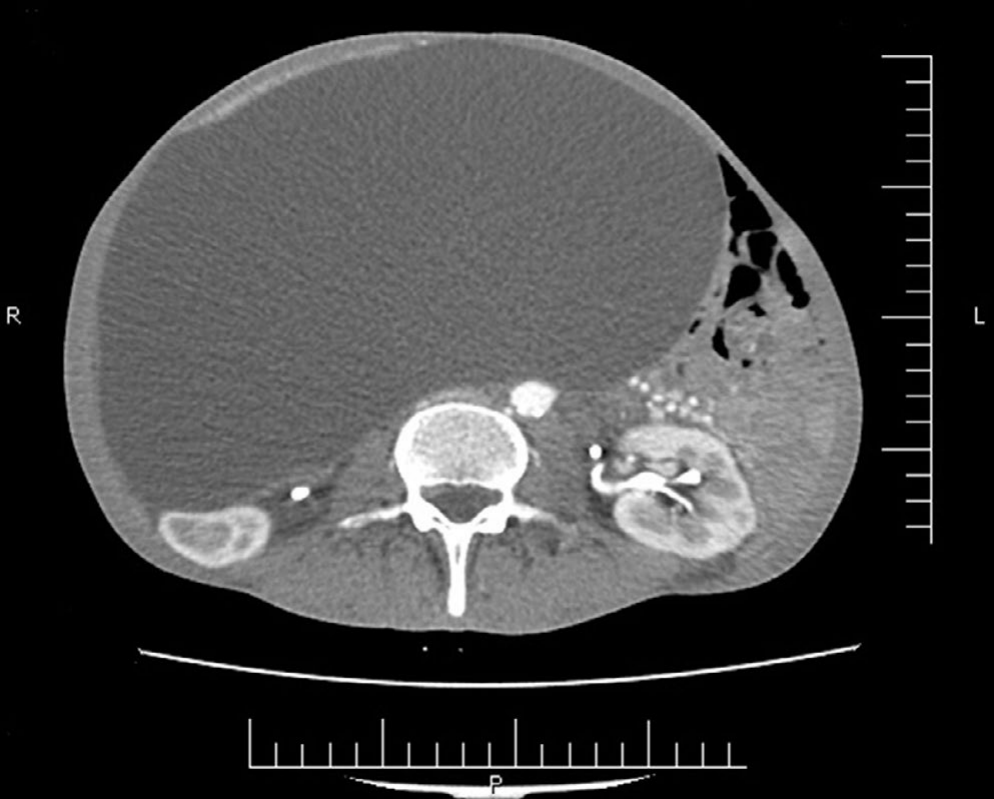
Axial abdominal computed tomography scan demonstrated a huge hypodense mass occupying the abdominal cavity and compressing intestinal loops

**FIGURE 2:**
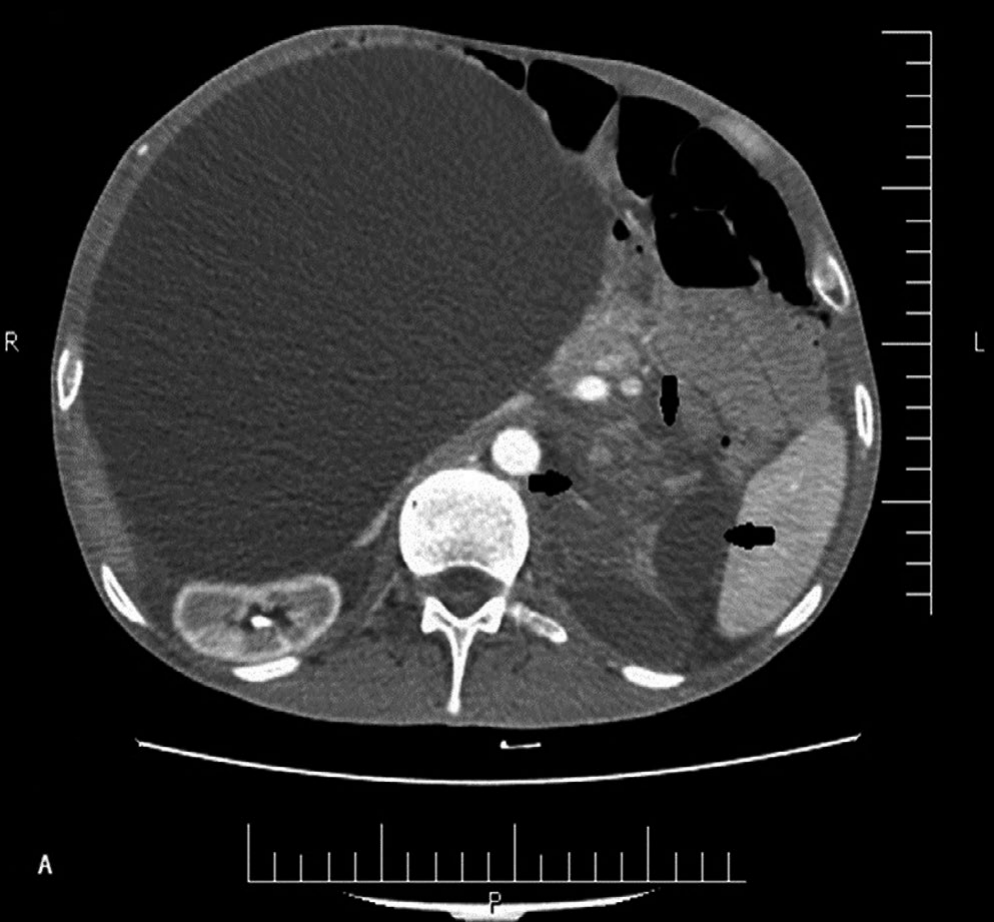
Abdominal computed tomography scan depicted retroperitoneal heterogeneous mass (black arrows)

Computed tomography scan (CT scan) depicted a 21 cm × 33 cm nonenhancement monolocular cyst in the right part of the abdomen (Figure 1). Furthermore, another mass sized 67 mm × 93 mm was observed in the left adrenal gland (Figure 2). The cystic mass exerted pressure on intestinal loops and moved them toward left. Although the patient had no significant symptom of pheochromocytoma, essential examinations were conducted in order to rule out pheochromocytoma. The results showed that 24‐hour urine metanephrine, vanillylmandelic acid, and catecholamine were in the normal range. The patient had become a candidate for surgery at the surgeon's discretion. However, he was not mentally in a proper condition to decide; therefore, his legal guardians were required to sign satisfaction letters. According to the psychiatrist's recommendation, the patient's drugs were replaced with injectable ones before the operation; then, he underwent a midline laparotomy operation. In intraoperative observation, the entire abdomen was occupied by a giant cystic mass. The mass was mobile and not adhere to the adjacent organs. The cyst was gently resected from the ascending colon mesentery while maintaining the mesenteric vessels. Afterward, left adrenalectomy was carried out with the same approach. The pathology report revealed that the abdominal mass consisted of a large cystic thin‐wall mass with smooth surface and sized 33 cm × 18 cm × 23 cm. Opening of the cyst revealed 5 liter yellowish clear fluids.

**FIGURE 3:**
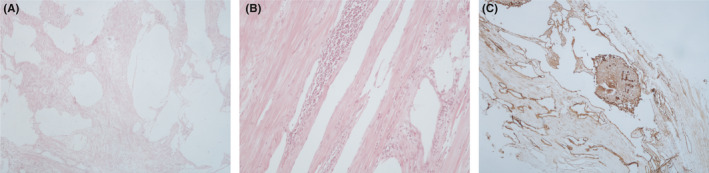
Histopathological characteristics of cyst. A, Various sized cystic and dilated vascular spaces which separated by a hypocellular stroma, formed by smooth muscle cells (H&amp;E, ×45). B, Patchy aggregation of small lymphocytes in the wall of vascular spaces (H&amp;E, ×100). C, Immunostaining for D2‐40 highlights lymphatic spaces (immunostaining D2‐40, ×100)

**FIGURE 4:**
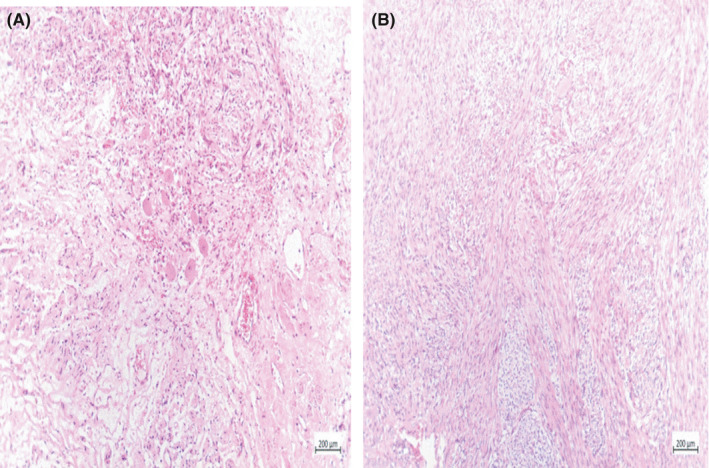
Histopathological characteristics of adrenal mass. A, Left hand: ganglioneuroma of adrenal gland showing clusters of mature ganglion cells surrounded by the Schwannian stroma. B, Right hand: ganglioneuroma of adrenal gland showing fascicles of the Schwann cells. (hematoxylin and eosin stain, original magnification ×200)

The adrenal mass consisted of three fragments of brown‐gray soft to rubbery tissue. On cut sections, a firm grayish creamy homogenous encapsulated lesion with irregular border with the size of 3 cm × 2.5 cm × 2 cm was noted. Histopathological examinations reported that the abdominal and adrenal resected masses were lymphangioma cyst and ganglioneuroma, respectively (Figure 3, Figure 4A,B).

The postoperation recovery was uneventful, and the postoperation conditions of the patient, including the elimination of the obstruction symptoms, abdominal distension, and tenderness were indicators of a satisfactory surgery. After 5 days, the patient was discharged. In the 4‐year follow‐up, the patient gained 30 kg of weight, and there were no signs and symptoms of recurrence of the masses.

## 3 DISCUSSION

Mesenteric cyst is a rare abdominal tumor with an incidence rate of 1 per 100 000 adults. It is observed that the gender of patients has no effect in its occurrence. Mesenteric cyst can be diagnosed anywhere from the small intestine to the rectum. In 66% of cases, it has been found in the small intestine (mainly arose from ileum mesentery), and in 33% of cases, it has been in the colon mesentery (mostly arose from the right colon).^2^

Although the exact etiology of these masses is unknown, it is believed that factors such as benign proliferation of ectopic lymphatics, abdominal trauma, lymphatic obstruction, and degeneration of some lymph nodes may be involved in forming MCs.^1^

There is no specific pathognomic symptom for MCs. Depending on the expansion and location of the tumor, there may be a spectrum of symptoms ranging from no symptoms and random diagnosis in radiographic investigation to symptoms including abdominal mass, vomiting, obstipation, and abdominal distension. Furthermore, it may express chronic signs such as anemia and weight loss.

In the case of late intervention, MCs may lead to some complications such as rupture, volvulus, obstruction, and torsion. In these scenarios, an emergent surgery would be the best approach.^5^, ^6^

Differential diagnosis of MCs are (a) cysts with lymphatic origin such as simple lymphatic cyst or lymphangioma, (b) cysts with mesothelial origin such as simple mesothelial cyst, and benign or malignant cystic mesothelioma, (c) enteric cysts such as enteric duplication cyst, (d) cysts with urogenital origin, (e) dermoid cysts such as mature cystic teratoma, and (f) nonpancreatic pseudocysts such as traumatic and infectious cysts.^7^ In order to differentiate mesenteric and omental masses, Tillaux suggested that mesenteric masses are characterized by their horizontal movement, while omental masses move in all directions. This feature is known as Tillaux's sign.^6^

Computer tomography (CT) and ultrasound (US) can be effective tools in the diagnosis of abdominal masses. Magnetic resonance imaging (MRI) is also helpful in diagnosis. US is employed in order to differentiate cystic from solid masses and determine the origin of mass. CT is also a useful tool in differentiating simple cysts (monolocular cysts) from multilocular cysts. Moreover, the origin of the cyst and its relation with the surrounding organs can be displayed in CTs. MRIs are more accurate compared with CTs and can provide more details; however, in most cases, CTs are sufficient for making an accurate diagnosis.^8^, ^9^

The best treatment option for these cysts is either complete resection of the mass with or without the mesenteric origin or, in some cases, segmental resection of the involved intestine. This operation lowers the risk of malignancy transformation and can be performed through either laparoscopic or open surgery. In open surgeries, 5‐8 days of postoperation hospitalization is required, while in laparoscopic surgery 1 or 2 days is enough. However, in the larger sizes of these masses, the possibility of the laparoscopic surgery is limited. Furthermore, when the patient is not a good candidate for surgery, marsupialization, internal drainage, and aspiration are considered as suitable alternatives. However, the possibility of recurrence, infection, and fistulization increases.^3^, ^9^, ^10^

The second tumor, GN, is a benign tumor originating from neural crest cells, and its incidence rates in retroperitoneum and posterior mediastinum are 32%‐52% and 39%‐43%, respectively. However, in some rare cases, they might be found in adrenal medulla.

Ganglioneuromas present no pathognomonic symptoms and often are hormonally silent. They will cause no signs in patients, unless they are giant tumors and compress the surrounding organs or they are endocrinologically active. However, none of these symptoms are specific to GNs.^4^

In sonography, these tumors appear as homogenous and hypoechogenic lesions. In CT, they are revealed as solid homogenous and hypodense masses. In MRI, T1‐weighted images usually show a lobulated homogenous hypointense mass, and T2‐weighted images show a heterogeneous one. However, none of these preoperational investigations can lead to a definite diagnosis. In some cases, these tumors cause an increase in urinary or plasma catecholamine or an increase in the level of steroid hormones such as cortisol and aldosterone. Nevertheless, these signs are not specific in diagnosis and sometimes cause misdirection. In fact, a definite diagnosis can only be made through postoperation histopathological examinations.^5^ Therefore, general approach to these tumors is performing a surgery that can be either open or laparoscopic. As mentioned, in tumors larger than 7 cm, the possibility of laparoscopic surgery is limited.

There is a little probability of malignancy in GNs; therefore, complete resection of the mass is required. It is not necessary to continue the treatment with adjuvant therapy; in other words, GNs have a good prognosis.^11^

In conclusion, computed tomography scan and magnetic resonance imaging are both employed in the diagnosis, and the results are verified with the pathology report. It is noted that both of these masses required to be excised through either open or laparoscopic surgery, depending on the size of the tumor.

The probability of random formation of the two tumors of ganglioneuroma and lymphangioma in a schizophrenic patient is almost zero, and it seems that some kind of syndrome is the causative factor. This probability encouraged us to search for reports of a syndrome along with probable simultaneous occurrence of these two tumors in the literature. Eventually, the only conceivable hypothesis was the involvement of the Noonan syndrome in which ganglioneuroma or lymphangioma might be observed.^12^ Additionally, there was a report on schizophrenia in one of the Noonan syndrome cases.^13^ Therefore, related genetic tests were recommended to diagnose the Noonan syndrome. However, due to the patient's disapproval of the tests, this possibility remained a mere hypothesis.

This patient was a known case of schizophrenia for several years. On the other hand, pseudocyesis is reported as a rare syndrome in schizophrenic patients.^14^, ^15^, ^16^, ^17^ Thus, symptoms such as vomiting and belly expansion in this patient were attributed to pseudocyesis syndrome. However, with the appearance of bowel obstruction symptoms and severe abdominal distension, the psychiatrist questioned pseudocyesis syndrome.

In further investigations, a GMC was detected, whose extreme size was rare, considering its benign nature. Moreover, during radiological examinations a ganglioneuroma was observed in adrenal which is also known as an adrenal incidentaloma.

## 4 CONCLUSION

In conclusion, it is advised that in psychological patients, somatically expressed the symptoms which imply another psychological syndrome should be dealt with cautiously.

## CONFLICT OF INTEREST

There is no conflict of interest to declare.

## AUTHOR CONTRIBUTIONS

AN: contributed to study concept and design and critically revised the manuscript for important intellectual content. BB and AS: acquired data, performed histopathological examinations, and reported images. FJ: drafted the manuscript and supervised the study. All authors: read and approved the final manuscript.

## ETHICAL APPROVAL

The present study is in compliance with ethical standards and standards of research involving humansAUTHOR: 'Ethical statement' has been changed as 'Ethical approval'. Check and confirm.

## CONSENT FOR PUBLICATION

Because the patient was not mentally in a proper condition to sign the informed consent, therefore, his legal guardians signed the form for publication of this case report and any accompanying images. A copy of the written consent is available for review by the Editor‐in‐Chief of this journal.

## Data Availability

Data in the current study are available from the corresponding author on reasonable request.
